# Danger Signals and Graft-versus-host Disease: Current Understanding and Future Perspectives

**DOI:** 10.3389/fimmu.2016.00539

**Published:** 2016-11-29

**Authors:** Tomomi Toubai, Nathan D. Mathewson, John Magenau, Pavan Reddy

**Affiliations:** ^1^Division of Hematology and Oncology, Department of Internal Medicine, University of Michigan Comprehensive Cancer Center, Ann Arbor, MI, USA; ^2^Department of Cancer Immunology and Virology, Dana-Farber Cancer Institute, Boston, MA, USA

**Keywords:** allogeneic hematopoietic stem cell transplantation, graft-versus-host-disease, danger signals, pathogen-associated molecular patterns, damage-associated molecular patterns

## Abstract

Graft-versus-host response after allogeneic hematopoietic stem cell transplantation (allo-HCT) represents one of the most intense inflammatory responses observed in humans. Host conditioning facilitates engraftment of donor cells, but the tissue injury caused from it primes the critical first steps in the development of acute graft-versus-host disease (GVHD). Tissue injuries release pro-inflammatory cytokines (such as TNF-α, IL-1β, and IL-6) through widespread stimulation of pattern recognition receptors (PRRs) by the release of danger stimuli, such as damage-associated molecular patterns (DAMPs) and pathogen-associated molecular patterns (PAMPs). DAMPs and PAMPs function as potent stimulators for host and donor-derived antigen presenting cells (APCs) that in turn activate and amplify the responses of alloreactive donor T cells. Emerging data also point towards a role for suppression of DAMP induced inflammation by the APCs and donor T cells in mitigating GVHD severity. In this review, we summarize the current understanding on the role of danger stimuli, such as the DAMPs and PAMPs, in GVHD.

## Introduction

Allogeneic hematopoietic stem cell transplantation (allo-HCT) has become widely used as a curative therapy for a variety of life-threatening hematological malignancies and congenital immune deficiencies (1). However, graft-versus-host disease (GVHD) remains as significant obstacle to improving the success of this treatment (2). The cause of GVHD reflects a complex process involving immune dysregulation in the context of recovering immunocompetent donor cells in recipients of allo-HCT. Donor T cells play a central role in the pathogenesis of acute GVHD. However, emerging data in the past 15 years have demonstrated a key role for donor, or recipient antigen presenting cells (APCs), derived from both hematopoietic and non-hematopoietic cells. Although current strategies of the prevention and treatment of acute GVHD mainly target T cells, modulating APC function represents a promising additional strategy for reducing acute GVHD. Therefore, a greater understanding of how APCs are activated and regulated is of significant interest. Myeloablative or reduced intensity conditioning regimens are a prerequisite for facilitating engraftment of donor hematopoietic cells, and for eliminating residual tumor cells, but they also cause significant host tissue damage. The impact of damage responses on APCs has become an active area of research. Host tissue injuries by conditioning regimens release “danger signals” including pathogen-associated molecular patterns (PAMPs), such as lipopolysaccharides (LPS), and damage-associated molecular patterns (DAMPs), such as high mobility group box 1 (HMGB-1) as well as pro-inflammatory cytokines, such as interleukin (IL)-1β, IL-6, and tumor necrosis factor (TNF)-α from the inflamed tissues. These danger signals activate host or donor APCs that in turn present allo-antigens *via* major histocompatibility complex (MHC) class I or class II to donor T cells. In addition, activated APCs produce an abundance of T-cell stimulating cytokines, such as IL-12, which further escalate the inflammatory response. In this review, we describe several encouraging investigations that have been conducted in both experimental bone marrow transplantation (BMT) models and humans over the last two decades. We further summarize the updated findings of how DAMPs and PAMPs amplify or mitigate GVHD and explore potential new strategies for the regulation of these “danger signals” in the regulation of GVHD.

## Danger Signals in GVHD Development

**Figure 1:**
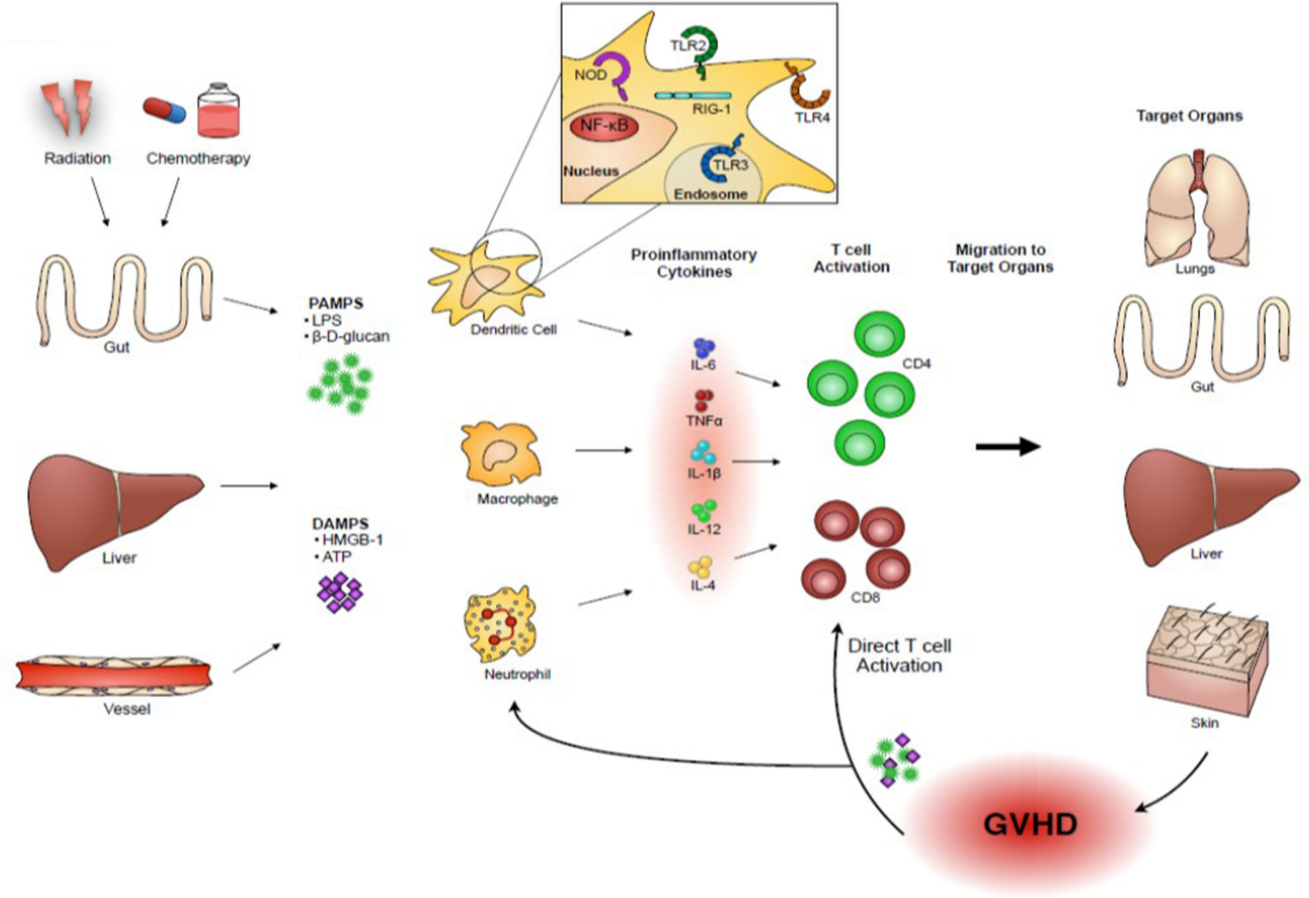
**Danger signals play an important role in acute GVHD pathogenesis**. Host tissue injuries by conditioning regimens release “danger signals” including pathogen-associated molecular patterns (PAMPs), such as lipopolysaccharides (LPS) and β-D-glucans, and damage-associated molecular patterns (DAMPs), such as high mobility group box 1 (HMGB-1) and adenosine triphosphate (ATP). These danger signals activate host or donor antigen-presenting cells (APCs), such as dendritic cells and macrophages, which in turn present alloantigens *via* major histocompatibility complex (MHC) class I or class II to donor T cells. In addition, activated APCs produce an abundance of pro-inflammatory cytokines, such as interleukin (IL)-1β, IL-6, and tumor necrosis factor (TNF)-α, and T-cell stimulating cytokines, such as IL-12, which further escalate the inflammatory response. Activated donor T cells proliferate and differentiate into effector T cells that migrate to target organs and cause GVHD. Upon target tissue destruction, additional PAMPs and DAMPs are released and they might perpetuate GVHD responses.

PAMPs are non-host derive molecules derived from microbes and are recognized by pattern recognition receptors (PRRs) that initiate and sustain the innate immune responses for protecting host from foreign pathogens (3). DAMPs are host-derived molecules released by host tissue damages and binds to PRRs that initiate and sustain non-infectious immune responses (4). These DAMPs and PAMPs are released as a consequence of conditioning-related tissue damage after allo-HCT. They activate APCs that in turn stimulate donor T cell proliferation and differentiation into effector T cells that migrate to target organs and cause GVHD. Upon target tissue destruction, additional PAMPs and DAMPs are released that perpetuate and amplify GVHD (Figure 1). Therefore, our understanding of the release of PAMPs/DAMPs and ways to limit this potentially lethal immunologic cascade by ameliorating tissue damages by inhibiting danger signaling with specific inhibitors may be important for mitigating the intensity of GVHD.

## Role of Specific PRRs in GVHD

Danger signaling is transmitted through PRRs when they bind PAPMs and DAMPs. Several signaling pathways, such as toll-like receptor (TLR), Nucleotide-binding oligomerization domain (NOD)-like receptor (NLR), and retinoic acid-inducible gene 1 (RIG-I) signaling, are recognized. The detailed mechanisms are recently reviewed in several articles (5–7). In this review, we focus on some of these receptors that have been implicated in GVHD.

## Toll-Like Receptors

Toll-like receptors are one of the PRRs and play a key role in innate immune responses by recognizing PAMPs as well as DAMPs (8). TLRs are expressed on a variety of cells derived from both hematopoietic and non-hematopoietic lineages (8). We discuss below the experimental studies of TLRs in the pathogenesis of acute GVHD. The studies are also summarized in Table 1.

**Table 1:** **The role of TLRs in the pathogenesis of acute GVHD**.

BMT models	MHC	Conditioning	Donor cells	Results	Reference
**TLR4**					

B6 (H2^b^) → C3H/HeJ (H2^k^) (TLR4 mutant)	Mismatch	9 Gy	BM: 5 × 10^6^	GVHD: ↑	(9)
			SP: 2.5 × 10^7^		

BALB/c (H2^d^) → B6-TLR4^−/−^ (H2^b^)	Mismatch	10.5 Gy	BM: 1 × 10^7^	GVHD: →	(10)
			SP: 2 × 10^7^		

B6-TLR4^−/−^ (H2^b^) → BALB/c (H2^d^)	Mismatch	9 Gy	BM: 1 × 10^7^	GVHD: ↓	(10)
			SP: 2 × 10^7^		

C3H/Hej (H2^k^) (LPS resistant) → (C3FeB6)F1 (H2^b/k^)	Mismatch, haploidentical	11 Gy	TCD-BM: 5 × 10^6^	GVHD: ↓	(11)
			Tcells: 0.25–1 × 10^6^		

BALB/c (H-2^d^) → B6-TLR2/4^−/−^ (H-2^b^)	Mismatch	Treosulfan + cyclophosphamide	BM: 5 × 10^6^	GVHD severity: ↓, mortality: →	(12)
			SP: 3 × 10^6^		

129S6 (H2b) → B6-TLR4^−/−^ (H2^b^)	Match, multiple minor antigen mismatch	11 Gy	BM: 5 × 10^6^	GVHD: →	(13)
			SP: 30 × 10^6^		

B6-TLR4^−/−^ (H2^b^) → 129 *Rag2*^−/−^ (H2^b^)	Match, multiple minor antigen mismatch	Anti-NK11 or anti-asialoGM1, ±7 Gy	SP: 30 × 10^6^	GVHD: ↓	(13)

BALB/c (H2^d^) → B6-TLR4^−/−^ (H2^b^)	Mismatch	9 Gy	BM: 1 × 10^7^	GVHD: →	(14)
			SP: 4–5 × 10^7^		

C3Hsw (H2^b^) → B10ScNcr-TLR4^−/−^ (H2^b^)	Match, multiple minor antigen mismatch	10 Gy	BM: 1 × 10^7^	GVHD: →	(15)
			CD8^+^ T cells: 2 × 10^6^		

**MyD88/TRIF**					

BALB/c (H2^d^) → B6-MyD88^−/−^ (H2^b^)	Mismatch	Treosulfan + cyclophosphamide	BM: 5 × 10^6^	GVHD severity: ↓, mortality: →	(12)
			SP: 3 × 10^6^		

B6-MyD88^−/−^ (H2^b^) → B6D2F1 (H2^b/d^)	Mismatch, haploidentical	11 Gy	TCD-BM: 5 × 10^6^	GVHD: ↑	(16, 17)
			Tcells: 1–2 × 10^6^		

B6-MyD88^−/−^ (H2^b^) → 129 *Rag2*^−/−^ (H2^b^)	Match, multiple minor antigen mismatch	Anti-NK11 or anti-asialoGM1, ±7 Gy	SP: 30 × 10^6^	GVHD: ↓	(13)

BALB/c (H2^d^) → B6-TRIF^−/−^ (H-2^b^)	Mismatch	Treosulfan + cyclophosphamide	BM: 5 × 10^6^	GVHD severity: ↓, mortality: →	(12)
			SP: 3 × 10^6^		

C3Hsw (H2^b^) → B6 LPS2 (TRIF^−/−^)(H2^b^)	Match, multiple minor antigen mismatch	10 Gy	BM: 1 × 10^7^	GVHD: →	(15)
			CD8^+^ T cells: 2 × 10^6^		

B6-TRIF^−/−^ (H2^b^) → 129 *Rag2*^−/−^ (H2^b^)	Match, multiple minor antigen mismatch	Anti-NK11 or anti-asialoGM1, ±7 Gy	SP: 30 × 10^6^	GVHD: →	(13)

**TLR2**					

B6-TLR2^−/−^ (H2^b^) → B6D2F1 (H2^b/d^)	Mismatch, haploidentical	11 Gy	TCD-BM: 5 × 10^6^	GVHD: →	(20)
			SP: 2 × 10^7^		

B6-TLR2^−/−^ (H2^b^) → BALB/c (H2^d^)	Mismatch	85 Gy	TCD-BM: 5 × 10^6^	GVHD: →	(20)
			SP: 2 × 10^7^		

**TLR5**					

B10BR (H2^k^) → B6 (H2^b^) with flagellin (50 μg)	Mismatch	11 Gy	TCD-BM: 5 × 10^6^	GVHD: ↓	(28)
			SP: 5 × 10^6^		

**TLR9**					

B6 (H2^b^) → B10BR (H2^k^) with CpG (100 μg)	Mismatch	8 Gy	BM: 5 × 10^6^	GVHD: ↑	(40)
			SP: 25 × 10^6^		

BALB/c(H2^d^) → B6 (H2^b^) with CpG (100 μg)	Mismatch	8 Gy	BM: 5 × 10^6^	GVHD: ↑	(40)
			SP: 15 × 10^6^		

BALB/c (H2^d^) → B6 (H2^b^) with CpG (50–100 μg)	Mismatch	10 Gy	BM: 5 × 10^6^	GVHD: ↑	(41)
			SP: 1 × 10^7^		

BALB/c (H2^d^) → B6-TLR9^−/−^ (H2^b^)	Mismatch	9 Gy	BM: 1 × 10^7^	GVHD: ↓	(14)
			SP: 4 × 10^7^		

BALB/c (H-2^d^) → B6-TLR9^−/−^ (H-2^b^)	Mismatch	Treosulfan + cyclophosphamide	BM: 5 × 10^6^	GVHD: ↓	(12)
			SP: 3 × 10^6^		

**TLR3**					

C3Hsw (H2^b^) → [TLR3^−/−^ (H2^b^) → B6 (H2^b^)]	Match, multiple minor antigen mismatch	9 Gy	TCD-BM: 5 × 10^6^	GVHD: →	(48)
			CD8^+^ T cells: 0.5 × 10^6^		

### TLR4

TLR4 is a cell-surface receptor for PAMPs such as LPS and also for DAMPs. TLR4 is broadly expressed on many immune cells, such as dendritic cells (DCs). TLR4 signaling is transmitted through intracellular adaptor molecules myeloid differentiation primary response gene 88 (MyD88) and Toll/IL-1receptor (TIR)-domain-containing adaptor-inducing interferon β (TRIF) that activate NF-κB signaling that potently enhances expression of pro-inflammatory cytokines. The role of TLR4 in APCs for mediating acute GVHD remains controversial. Mutations in TLR4 are involved in the reduction of GVHD responses by hyporesponsiveness of APCs to LPS stimulation while over activation of TLR4 signaling results in exacerbation of GVHD (9). When TLR4^−/−^ animals were used as either donor or recipient, acute GVHD severity and mortality were significantly ameliorated in MHC-mismatched B6 into BALB/c model by altering DC functions in TLR4^−/−^ APCs (10). This finding was consistent with a previous report in which recipients receiving LPS resistant donor cells demonstrated less GVHD and prolonged survival in MHC-mismatched haploidentical BMT (11). TLR2/4^−/−^ animals receiving MHC-mismatched BMT (BALB/c into B6) also showed significantly less intestinal GVHD, but reduction was appeared dependent on conditioning intensity (12). In minor mismatched BMT context, MyD88-mediated TLR4 signaling on donor, but not recipient cells, was required for mediating acute GVHD (13). In addition, when TLR4 signaling was impaired in host APCs, that is, in the case of the absence of MyD88, TRIF, or both MyD88 and TRIF expression, acute GVHD severity and mortality were equivalent to WT animals (14, 15). While there is an increasing understanding of the key role of TLR4 signaling in contributing to the initiating event of GVHD, such disparate findings indicate that the role of TLR4 signaling for mediating GVHD may differ depending on the strain, the cell type where TLR4 is mutated, and the conditioning.

### MyD88/TRIF

As MyD88 is required for the signaling of many TLRs, when MyD88^−/−^ animals were used as the host, acute GVHD was significantly improved (12). In contrast, the recipients that received MyD88^−/−^ T cell depleted BM (TCD-BM) cells showed greater intestinal GVHD (16) but reduced hepatic GVHD. This was found to be dependent on myeloid-derived suppressor cells (MDSCs) (17). In addition, MyD88^−/−^ donor T cells reduced graft-versus-tumor (GVT) activity through the expansion of Foxp3- and IL-4-producing T cells in MHC-mismatched haploidentical B6 into B6D2F1 model (18). However, donor MyD88 was shown to be required in minor mismatch GVHD (13). TRIF is also required to transmit TLR signaling, but its role seems to be negligible in the development of GVHD (12, 13, 15). Collectively, these studies suggest that MyD88 may have pleiotropic functions that are cell intrinsic during allo-HCT.

### TLR2

TLR2 is a cell-surface receptor expressed on APCs as well as T cells. TLR2 recognizes cell-wall components such as peptidoglycan (PGN) from gram-positive bacteria as well as zymosan from yeast. Intriguingly, granulocyte-colony stimulating factor (G-CSF) mobilized donor grafts showed the increase level of TLR2 expression on myeloid cell populations (19), but upregulated TLR2 expression did not correlate with enhanced allogeneic responses (20). The studies utilizing TLR2^−/−^ animals as either donor or host demonstrated that TLR2 has little effect on acute GVHD (12, 20).

### TLR5

TLR5 recognizes flagellin that is an essential component of bacterial flagella from both gram-negative and -positive bacteria and regulates immunity (21–26). CBLB502, a TLR5 agonist and a polypeptide drug derived from Salmonella flagellin, protects intestinal and hematopoietic cells from total body irradiation (TBI) in mice and primates (27). Consistent with this observation, when TLR5 agonist was administered before conditioning, acute GVHD was reduced with enhanced anti-CMV immunity in both MHC-mismatched and haploidentical murine models (28, 29). By contrast, TLR5 mRNA expression on peripheral blood, especially in the Lin(−)HLADR(−)CD33(+) CD16(+) and CD14(++)CD16(−) monocytes, was increased in the patients with GVHD after receiving adaptive Treg infusion for prevention of GVHD in human (30).

### TLR7

TLR7 recognizes endosomal single strand ribonucleic acids (RNAs) leading to production of type I interferons (IFNs), pro-inflammatory cytokines, as well as regulatory cytokines (31, 32). TLR7 is critical for antiviral immunity and the development of autoimmune diseases (33–36). The contribution of TLR7 to acute GVHD is not well characterized. TLR7 ligand imiquimod was shown to increase alloreactivity of host-derived DCs and Langerhans cells (LCs) in the skin and to enhance donor lymphocyte infusions (DLIs)-mediated GVHD in MHC-matched multiple minor antigen-mismatched model of BMT (37). Because Type I IFNs are indispensable in the antitumor responses, whether TLR7 agonists increase GVT activities without enhancing GVHD after allo-HCT is of significant interest.

### TLR9

TLR9 recognizes unmethylated cytosine-phophorothionate-guanine (CpG) dinucleatides in the bacterial DNA and triggers a Th1-mediated inflammatory response (38). CpG-mediated immune responses through TLR9 distinguish bacterial DNA from self-DNAs. TLR9 is expressed intracellularly in both immune and non-hematopoietic derived cells, such as endothelial and epithelial cells (39). Administration of CpG oligonucleotides (CpG ODNs), a synthetic TLR9 ligand, exacerbates acute GVHD in a host APC and IFN-γ dependent manner (40, 41). In addition, CpG ODNs enhances rejection donor HSCs in donor-derived APC dependent manner (40). When lethally or sub-lethally conditioned TLR9^−/−^ animals were used as hosts, acute GVHD severity and mortality was ameliorated. This was dependent on the expression of TLR9 expression on the non-hematopoietic cells (12, 14). However, clinical studies exploring TLR9 polymorphisms in allo-HCT hosts suggested that those with homozygous CC gene variant of TLR9 (which correlates with lower expression of TLR9 mRNA) showed significantly improved overall survival (OS) and reduced relapse rate with no difference in acute GVHD when compared with patients having TC/TT gene variants (42, 43). Donor TLR9 gene tag single nucleotide polymorphisms (SNPs), +1174A/G (rs352139) and +1635 C/T (rs 352140), respectively, correlated increased severity of acute GVHD and CMV reactivation (44).

### TLR3

TLR3 recognizes double-stranded RNA (dsRNA), which is produced by most of viruses. It signals through interferon regulatory factor (IRF)3 and activates NF-κB and enhances production of type I IFNs (45, 46). TLR3 also plays an important role in enhancing antigen presentation in APCs (47). Using chimeric recipients with TLR3^−/−^ hematopoietic cells, we have already demonstrated that TLR3 deficiency in host APCs showed equivalent GVHD severity and mortality to WT animals but impaired GVT activity in MHC-matched multiple minor mismatched BMT model. Activation of TLR3 by polyinosine-polycytidylic acid (Poly I:C) improved GVT activity without enhancing GVHD (48). Because type I IFNs are essential role in antitumor immune responses (49, 50), TLR3 may have greater influence on GVT activity.

### NLR Signaling

Nucleotide-binding oligomerization domain-like receptors are subtype of PPRs that function as cytoplasmic sensors of PAMPs and DAMPs. NLRs are expressed by majority of immune cells and some non-immune cells. NLRs have been extensively studies for their role in innate immunity. NOD1 and NOD2 are the most widely investigated NLRs in GVHD. NOD1 and NOD2 recognize different kinds of PGN fragments from bacterial cell wall. NOD1 binds to diaminopimelate-containing N-acetyl glucosamine-N-acetylmuramic acid (GluNAc-MurNac) tripeptide from gram-negative bacterial PGN (51, 52), while NOD2 binds to muramyl dipeptide (MDP) that is produced by all bacteria (53). Once NOD1 and NOD2 are ligated, NF-κB and mitogen-activated protein kinase (MAPK) pathways are activated through the caspase recruitment domain (CARD)-containing serine/threonine kinase, receptor-interacting protein 2 (Rip2) (54), and induce pro-inflammatory cytokines. NOD1 and NOD2 signaling is also involved in endoplasmic reticulum (ER) stress induced inflammation through the IRE1α/TRAF2 signaling pathway (55) and production of antimicrobial peptides in the intestinal tract (56). The role of NOD2 signaling pathway in allo-HCT is somewhat controversial. NOD2 polymorphism in both donor and recipient was associated with increased transplant-related mortality in humans after HLA-identical sibling HCT or T cell depleted HCT as well as increased GVHD severity (43, 57–64). However, other studies demonstrated that NOD2 had no impact on outcome including GVHD severity and mortality (44, 65–69). In addition, intriguingly, NOD2 polymorphism was associated with increased relapse of leukemia after unrelated HCT (70). In experimental models, NOD2^−/−^ recipient animals showed exacerbated GVHD severity and morality, particularly intestinal GVHD. This was due to host APC activation in experimental BMT (71). By contrast, donor NOD^−/−^ BM cells reduced GVHD-related mortality in MHC-mismatched haploidentical BMT model (72).

### Inflammasomes

Inflammasomes are multiprotein molecules that are in the cytoplasm of immune cells, such as APCs, as well as non-hematopoietic cells. They consist of an adaptor protein, apoptosis associated speck-like protein containing a caspase recruit domain (CARD) (ASC), which has pyrin domain (PYD) and CARD, pro-caspase 1, and certain receptor proteins, such as NLR family members (NLRP1, NLRP3, NLRC4, NLRP6, NLRP7, and NLRP12), the protein absent in melanoma 2 (AIM2) (73, 74). Once inflammasomes are activated, they produce inflammatory cytokines, specifically IL-1β and IL-18, and induce pyroptosis, a highly inflammatory form of programed cell death (73, 74). This is called canonical inflammasome pathway, which in contrast to the non-canonical pathway activates caspase 11 in mouse and caspases 4 and 5 in humans (75). The detailed molecular and activation pathways of inflammasomes have recently been summarized in excellent reviews (73, 74). Studies exploring the role of inflammasomes in acute GVHD have recently been published. NLRP3 activation by the intestinal commensal bacteria and uric acid released after conditioning, enhanced GVHD severity and mortality by increasing levels of caspase-1, IL-1β, and TH17 cells (76). Another mechanism of enhanced GVHD by NLRP3 was shown to be associated with microRNA-155 dependent host DC migration toward sites of ATP release (77). In addition, inflammasome activation in the inflammatory milieu ameliorated the immune suppressive function of MDSCs and exacerbated GVHD (78). Relevant to clinical translation, the addition of TBI or busulfan and cyclophosphamide (BU/CY) conditioning is capable of mediating NLRP3 activation in the liver and enhancing inflammation (79). As therapeutic strategy, inhibiting NLRP3 activation by nucleotide reverse transcriptase inhibitors decreased GVHD severity and mortality (80). Further, NLRP3 inflammasome in human CD4^+^ T cells promotes IFN-γ production and Th1 differentiation by enhancing caspase 1-dependent IL-1β secretion mediated through intracellular C5 activation (81). These data suggest that NLRP3 inflammasome contribute to the functional important mediators of GVHD: donor T cells, APCs, and non-hematopoietic cells in target tissue. Consistent with experimental models, donor polymorphisms in the NLRP3 inflammasome have been associated with outcomes after allo-HCT. TT genotype at rs10925027 in NLRP3 was associated with disease relapse and donor GG genotype at rs1043684 in NLRP2 was associated with non-relapse mortality (NRM) and OS. Also, patient AA genotype at rs5862 in NLRP1 was associated with NRM and OS after HLA-matched sibling HCT (82). We recently found that a related but distinct inflammasome, NLRP6, expressed in intestinal epithelial cells regulates innate immune responses and intestine homeostasis though the regulating normal commensal bacteria (83, 84). Absence of NLRP6 improved GVHD contrary to models of inflammatory bowel disease (IBD). Intriguingly, NLRP6^−/−^ animals showed enhanced mucin family protein MUC2 expression in epithelial cells after allo-BMT (85). These results suggest that depending on the context, NLRP6 may exert opposite effects in various inflammatory disorders.

### RIG-I Signaling

RIG-I, melanoma differentiation-associated gene 5 (MDA5), and laboratory of genetics and physiology 2 (LGP2) are known as RIG-I-like receptors (RLRs). RIG-I and MDA5 contain a DExD/H box RNA helicase domain and CARD, but LGP2 has no CARD-like domain. These receptors bind to intracellular dsRNA or ssRNA and trigger innate antiviral responses by producing type I IFNs (86–91). Therefore, RIG-I pathways also play an important role in PAMPs and DAMPs-mediated inflammatory responses. However, whether RIG-I pathways facilitate GVHD development is presently unknown. Preliminary study suggests RIG-I-induced type I IFNs promote the regeneration of intestinal stem cells during acute tissue damage may ameliorate GVHD severity with preserving GVL activities in mouse model (92, 93).

### C-Type Lectin Receptors

C-type lectin receptors (CLRs) are expressed on myeloid-derived APCs as soluble or transmembrane embedded proteins. They directly activate NF-κB through spleen tyrosine kinase (SYK) (94) or indirectly, by cooperating with other PRRs such as TLRs (95–97). Stimulation of CLRs promotes the production of pro-inflammatory cytokines, effector T cell differentiation into Th1 and Th17 (98). CLRs are divided into two groups; group 1 CLRs belong to the mannose receptor family and group 2 CLRs belong to the asialoglycoprotein receptor family which has subfamilies, the DC-associated C-type lectin1 (Dectin-1) and DC immunoreceptor (DCIR) subfamily including Dectin-2 (95). These recognize mannose, fucose, and glucan carbo-hydrate structures of bacteria, fungi. Both Dectin1 and Dectin2 activate NF-κB by enhancing SYK signaling through either the cytoplasmic immunoreceptor tyrosine-based activation motif (ITAM) in Dectin1 (94) or the ITAM-containing adaptor molecules, such as Fc receptor γ-chain (Fcr γ) or DAP12 in Dectin2 (99). Clinical studies have suggested that the incidence of acute GVHD increases with candida colonization in dectin1 gene dependent manner (100, 101). In murine model, α-mannan, which is a major component of fungal cell wall, mediated Th17 dependent pulmonary GVHD in a host dectin2 dependent manner (102).

## Role of Specific PAMPs and DAMPs Proteins in GVHD

Both exogenous and endogenous danger signal proteins are released from damaged tissues and abnormal intestinal microbial colonies after conditioning. In addition, blood stream infection (BSI) caused by gut translocation of colonized bacteria is another critical source of PAMPs after allo-HCT (103). Experimental data showed that individual PAMPs and DAMPs proteins can function either independently or cooperatively to initiate GVHD.

### Lipopolysaccharides

Lipopolysaccharides (endotoxin) are membrane component of many gram-negative bacteria representing one of the earliest and most investigated PAMPs in GVHD. The role of LPS in GVHD is complex and controversial. LPS translocation due to gastrointestinal (GI) tract damage is correlated with conditioning intensity (104) and shown to contribute to GVHD in select model systems. LPS activates APCs including DCs and macrophages (MFs) triggering production of pro-inflammatory cytokines, such as TNF-α, IL-1β and IL-6 (105, 106). These events contribute idiopathic lung injuries after allo-BMT (107). Persistent exposure of LPS precipitates pulmonary GVHD pathogenesis because recipient mice directly exposed to repeated inhaled LPS after allo-BMT showed pulmonary GVHD in hematopoietic donor-derived C-C motif ligand 2 (CCL2) and C-C motif receptor (CCR2) dependent manner (108, 109). In addition to host-derived cells, sensitivity to LPS in donor non-T cells has been suggested to be involved in GVHD severity (11). LPS is one of the ligands of TLR4, which plays a key role in innate immune responses (110), and its signaling is transmitted through the common MyD88 and TRIF pathway that can nuclear translocation of NF-kB to induce expression of inflammatory cytokine genes (111–113). TLR4 mutations lead to LPS hyporesponsiveness (110). As noted above, role of TLR4 and MyD88 in GVHD seems to depend on the model system.

### Flagellin

The recipient animals treated with flagellin before allo-HCT demonstrated reduced GVHD mortality and enhanced immune reconstitution with preservation of antiviral and GVT effects after allo-HCT (28). Modulating TLR5 functions with flagellin enhanced GVT without exacerbating GVHD in CD8^+^ T cell and NK cell dependent model (114). Tumor reactive T cells engineered to produce flagellin along with expression of a melanoma-specific antigen-augmented antitumor responses. Contrary to enhanced T cell-mediated antitumor responses, TLR5-dependent commensal bacteria promote tumor development by expanding MDSCs and dampen antitumor immunity in TLR5- and IL-6-dependent manner (115). In addition, mesenchymal stem cells (MSCs), pretreated by flagellin showed increased Foxp3 expression, enhanced IL-10 production, and suppressed GVHD (116). Interestingly, TLR5 stimulation with flagellin protects gut mucosal tissue from damages caused by irradiation (27, 117).

### Damage-Associated Molecular Patterns

#### HMGB1

HMGB1 is a ubiquitous DNA-binding nuclear protein of all eukaryotic cells, binds to nucleosome and regulates gene transcriptions (118). By contrast, HMGB1 plays an important role in initiating innate immune responses because endogenous HMGB-1 that is located in nucleus in resting cells is acetylated and is released from damaged tissues. HMGB-1 binds to TLRs (TLR2 or TLR4) or receptor for advanced glycation endproducts (RAGE) and activates NF-κB or MAPK signaling to produce pro-inflammatory cytokines in especially DCs or MFs (119–123). Therefore, extracellular HMGB1 functions as a DAMP. In addition, the inflammatory milieu with abundant IFN-γ, TNF-α, as well as LPS may promote further HMGB1 release from DCs and MFs (124). Inflammasome and Janus kinase (JAK)/signal transducer and activator of transcription (STAT) 1 pathways are involved in molecular mechanisms of HMGB-1 release, which requires its acetylation and translocation from nucleus to cytoplasm and released to extracellular space through unique protein releasing pathway, such as pyroptosis. Recent reports suggests that HMGB1 promotes not only immune suppressive function through the facilitating MDSCs proliferation in cancer (125) but also protection from tissue injury in IBD by regulating cellular autophagy and apoptosis (126). Patients with HMGB1 polymorphism, the 2351insT minor allele, showed reduced grade II to IV acute GVHD following myeloablative allo-HCT (127). Increased serum levels of HMGB1 were observed in acute GVHD patients and donors treated with granulocyte-colony-stimulating factor (G-CSF) (128, 129). Myeloablative conditioning such as TBI or cyclophosphamide + TBI also increased serum HMGB1 levels consistent with its function as a DAMP (130).

#### Adenosine Triphosphate

All cells generate adenosine triphosphate (ATP) as the primary energy source *via* glycolysis and oxidative phosphorylation (OXPHOS) that is stored within cytoplasm and mitochondria (131). Once cells are exposed to stress or injury, ATP is released from damaged cells and the concentration of ATP in extracellular space is increased. Released ATP binds to purinergic receptor families, such as P2X expressed on the hematopoietic and non-hematopoietic cells, and can function as a potent DAMP (132, 133). In GVHD, extracellular ATP is dramatically increased after TBI and binds to P2X7R on host APCs. After its ligation, host APCs expressed greater co-stimulatory molecules, such as CD80 and CD86, and enhanced stimulation of donor CD4^+^ T cells and production of IFN-γ, and decreased Tregs. These results were associated with reduction of STAT5 phosphorylation and enhanced GVHD (134). Another purinergic receptor, P2Y2, in host hematopoietic derived APCs was shown to enhance GVHD (135). Increased extracellular ATP is regulated by ecto-nucleotidases, such as CD39, which phosphohydrolyzes ATP to adenosine diphosphate (ADP) and adenosine monophosphate (AMP) and then dephosphorylate into adenosine by CD73, also known as ecto-5′-nucleotidase (136). In line with this, agonists of the adenosine receptors (AR) decreased GVHD (137). Loss of this regulatory mechanism by CD73^−/−^ T cells or in APCs exacerbated GVHD (138, 139). The recent study showed that inhibiting Notch 1 signaling by inducing expression of A2A receptor in CD73 dependent manner is a critical mechanism of Treg-induced GVHD suppression (140). The immunosuppression of BM-derived MSCs in GVHD was also shown to be partially dependent on CD73 activity (141).

#### Uric Acid

Uric acid is a metabolite of purine nucleotide and hyperuricemia is known to lead gout (142). Uric acid is also released from injured cells, stimulates DC maturation, activates CD8^+^ T cell cytotoxic functions, and is recognized as an endogenous DAMP (143). Recent data show that uric acid contributes to GVHD severity by stimulating with NLRP3 inflammasome (76). Patients with acute GVHD show a high level of serum uric acid during the pretransplantation period and the patients received a recombinant urate oxidase appeared to show significantly reduced GVHD in phase I study. These results are consistent with a study that blood uric acid homeostasis may be altered after allo-HCT by conditioning and using cyclosporine A (144). However, a recent study showed low serum level of uric acid was associated with GVHD severity (145).

#### Heat Shock Proteins

Heat shock proteins (HSPs) work as molecular chaperones that enhance protein folding and intracellular transportation (146). HSPs have been demonstrated the association with chronic inflammatory diseases as well as autoimmune disease (147, 148). HSPs bind to TLR2/4 and mediate inflammatory responses. In GVHD, expression of HSP70 in lymphoid and target organs is increased and correlated with severity in both human and experimental GVHD (149–151). HSP70 homogene polymorphism (+2763 A/A) was associated with the development of GVHD (152). Another study demonstrated that antibodies to HSP70 and HSP90 increased in the patients with GVHD after allogeneic peripheral blood stem cell transplantation (allo-PBMCT) (153). HSP90 expression is increased in activated T cells and facilitates effector function and survival in activated T cells (154). HSP90-specific inhibitor decreased allogeneic T cell responses *in vitro* (155), but the *in vivo* effects on GVHD were not studied.

#### Heparan Sulfate Proteoglycans

Heparan sulfate proteoglycans (HSPGs) are component of extracellular matrix and play fundamental role in cell development, metabolism, and immunity (156, 157). HSPGs are crucial role in enhancing innate immune responses by stimulating DCs to enhance production of pro-inflammatory cytokines through TLR4 pathway (158). HSPGs promote neutrophils recruitment into the site of inflammation (159, 160) while enhance neutrophil infiltration exacerbates GVHD (161). The serum level of syndecan-1, which is one of the HSPGs, and heparin sulfate are increased in patients with GVHD (162, 163). In experimental models, heparin sulfate activates TLR4 signaling on DCs and leads to enhanced DC maturation and allogeneic T cell proliferation and increased GVHD severity (163). On the other hand, the absence of syndecan-4, which is one of the HSPGs and a ligand of DC-HIL that functions as a co-inhibitory pathway of donor T cell immune responses worsened GVHD (164).

#### Alpha-Mannan

The alpha-mannan (α-mannan) is a component of fungal cell wall as a known DAMP. The α-mannan is recognized by dectin1 and dectin2, which is one of the CLRs and activates NF-κB signaling through SYK and produces pro-inflammatory cytokines and effector T cell differentiation (96). α-mannan stimulated macrophages through dectin2, enhanced Th17 differentiation, and worsened lung GVHD (102). Colonization of *candida species* exacerbated GVHD in clinical studies (100, 101).

## Therapeutic Strategies through Modulating Danger Signaling

“Danger signals” are indispensable role in initiating and developing acute GVHD. Regulating danger signal in an efficient manner in early phase of allo-SCT would ameliorate GVHD and have a great therapeutic strategy. Herein, we summarize potential therapeutic strategies for prevention and treatment of GVHD through modulating this signaling pathway. The studies are also summarized in Table 2.

**Table 2:** **Targeting danger signals to ameliorate GVHD**.

Drug	Function	Results of preclinical model	Results of clinical trials	Reference
PPADS	P2X7R antagonist	FVB → BALB/c: GVHD↓	Not tested	(134, 165)
Brilliant blue G (BBG)		B6 → BALB/c: GVHD↓		
		B6 → BALB/c: GVHD↓		

Apyrase	ATP diphosphohydrolase	FVB → BALB/c: GVHD↓	Not tested	(134)
		B6 → BALB/c: GVHD↓		

ATL146e	Adenosine A2A receptor agonist	B6 → B6D2F1: GVHD↓	Not tested	(137, 166)
ATL370		B6 → B6D2F1: GVHD↓		
ATL1223		B6 → BALB/c: GVHD↓		

Alpha-1 antitrypsin (AAT)	Serine protease inhibitor (targeting heparin sulfate, IL-32)	B10.D2 → BALB/c: GVHD↓	Phase I/II: GVHD↓ w less toxicity	(163, 167–169, 175, 176)
		C3H.sw → B6: GVHD↓		
		B6 → B6D2F1: GVHD↓		
		B6 → C3H.sw: GVHD↓		

CD24 fusion protein	CD24 agonist (Siglec-G agonist)	BALB/c → B6: GVHD↓	Phase IIa (to be initiated in 2016)	(181, 182)
		B6 → BALB/c: GVHD↓		

### Purinergic Receptor Antagonist

P2X7 receptor on APCs binds to extracellular ATPs, which released from damaged tissues by conditioning, and activates APCs to produce pro-inflammatory cytokines. Therefore, P2X7R plays a key role in DAMPs-mediated inflammatory responses. Systemic administration of the broad-spectrum P2X7R antagonist, pyridoxal-phosphate-6-azophenyl-2′,4′-disulfonic acid (PPADS), or an ATP diphosphohydrolase, apyrase attenuated GVHD by suppressing APC activation (134). Additionally, another P2X7R receptor antagonist, brilliant blue G (BBG) improved liver function by regulating the infiltration of donor MFs and neutrophils in liver and attenuated GVHD (165). Beside P2X7R antagonist, adenosine A2A receptor agonist, ATL146e, decreased GVHD severity by modulating T cell activation and Treg function in experimental model (137, 166). Whether modulating other purinergic receptors ameliorates GVHD in the context allo-HCT needs to be studied.

### Alpha-1 Antitrypsin

Serine protease inhibitor alpha-1 antitrypsin (AAT) attenuates GVHD by inhibiting HS, one of the DAMPs, and reducing HS mediated allogenic T cell responses in murine model (163). Clinical investigation demonstrates that the patients who have GVHD increased the serum level of HS after allo-HCT (163). In addition, we and others found that AAT attenuates GVHD with reducing serum levels of pro-inflammatory cytokines but increasing IL-10 levels by modulating function of donor and host APCs as well as altering the ratio of donor effector T cells to Tregs (167, 168). AAT also inhibits IL-32 activation mediated by proteinase-3, which is a neutrophil granule serine proteinase (169, 170). AAT homeostasis after allo-HCT may be important for regulating allogeneic responses because elevated AAT clearance in stool was correlated with the severity of GI-GVHD and steroid resistant GVHD (SR-GVHD) in some studies (171–173), but not others (174). Consistent with this, we and others have demonstrated that AAT treatment for SR-GVHD-improved GVHD manifestations without significant adverse effects or increased rates of infection in a multicenter prospective or single institution phase I/II study (175, 176). This data indicates that AAT may be a rational first-line therapy for SR-GVHD or other high risk GVHD, which is associated with high mortality. Although the mechanism how AAT suppresses SR-GVHD has not been clearly elucidated, these encouraging findings warrant further prospective, randomized, and multi-centered study.

### Siglec-G: A Potential Negative Signaling for DAMPs-Mediated Inflammation

**Figure 2:**
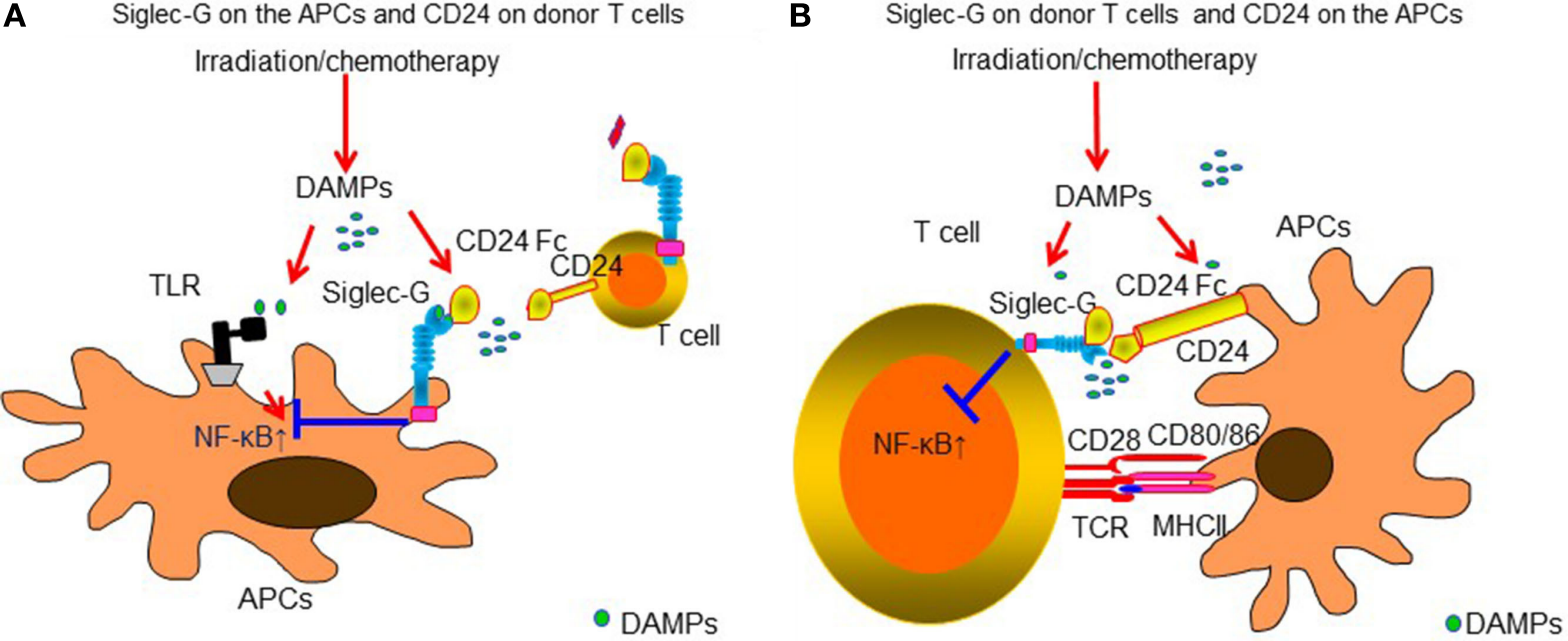
**Siglec-G-CD24 axis is critical for regulating acute GVHD**. Sialic acid-binding immunoglobulin-like lectins (Siglecs) have an immunoreceptor tyrosine-based inhibitory motifs (ITIM) or ITIM-like regions in their intracellular domains and negatively regulate DAMPs-mediated innate inflammatory responses. Siglec-G expression in host APCs plays an important role in protecting from DAMPs-mediated GVHD following conditioning-mediated tissue damage. Interaction of Siglec-G with CD24, a small glycosyl-phosphatidyl-inositol (GPI)-anchored glycoprotein on T cells that is recognized as a ligand of Siglec-G was critical for protection from GVHD. Enhancing Siglec-G-CD24 axis by a novel CD24 fusion protein (CD24Fc) mitigated GVHD **(A)**. In addition, enhancing the interactions between Siglec-G on T cells and CD24 on APCs with CD24Fc mitigated GVHD **(B)**.

Sialic acid-binding immunoglobulin like lectins (Siglecs) have an immunoreceptor tyrosine-based inhibitory motifs (ITIM) or ITIM-like regions in their intracellular domains and negatively regulate DAMPs-mediated innate inflammatory responses (177, 178) or induce B-cell tolerance by suppressing NF-κB pathways (179, 180). We observed that Siglec-G expression in host APCs plays an important role in protecting DAMPs-mediated GVHD following conditioning mediated tissue damage. Interaction of Siglec-G with CD24, a small glycosyl-phosphatidyl-inositol (GPI)-anchored glycoprotein on T cells that is recognized as a ligand of Siglec-G (181), was critical for protection from GVHD. Enhancing Siglec-G-CD24 axis by a novel CD24 fusion protein (CD24Fc), consisting of the extracellular domain of mature human CD24 linked to the human immunoglobulin G1 (IgG1) Fc domain, mitigated GVHD in multiple experimental BMT models (181). We also found that enhancing the interactions between Siglec-G on T cells and CD24 on APCs with CD24Fc mitigated GVHD while preserving GVT effects in experimental models as well as human PBMCs (182). The summarized mechanism of Siglec-G-CD24 axis for controlling GVHD is shown in Figure 2. Based on these preclinical studies, a prospective, randomized multi-centered phase IIa study is currently investigating whether the addition of CD24Fc to standard immune-prophylaxis can limit the incidence and severity of acute GVHD following myeloablative allo-HCT.

## Closing Remark

Danger signals mediate inflammatory responses through a multitude of PRRs that play a key role in the pathogenesis of GVHD. Once danger signals are released after conditioning, multiple innate immune signaling pathways are activated and amplified. Therefore, regulating danger signaling pathways in an effective manner is complex. Preclinical data suggest that targeting one specific signaling pathway or molecule may have only limited effects in reducing GVHD. In addition, the specific timing of regulation by blockade using antagonists may be a critical factor to consider. However, it is plausible that stimulating the negative regulating pathway that is commonly employed by several DAMPs may be a more rational way to mitigate GVHD. Thus exploring novel mechanisms of negative regulation of danger DAMP signaling that mediate lethal inflammatory responses should be carefully examined as new strategy of the prevention and treatment of GVHD. One potential benefit of regulating danger signaling is that GVT responses may be preserved due to selective attenuation of APCs with presumably limited effects on donor tumor-specific T cells necessary for mediating GVT responses. Clinical trials that investigate critical mediators of the danger response hold promise in the prevention of GVHD without affecting GVT responses.

## Author Contributions

The review article was designed and written by TT and PR. Both NM and JM helped with designing figures. together with the help of co-authors NM and JM. All of them performed literature review and critically discussed the published literature.

## Conflict of Interest Statement

The authors declare that the research was conducted in the absence of any commercial or financial relationships that could be construed as a potential conflict of interest.
